# Reply to ‘Are atrial human pluripotent stem cell-derived cardiomyocytes ready to identify drugs that beat atrial fibrillation?’

**DOI:** 10.1038/s41467-021-21950-6

**Published:** 2021-03-19

**Authors:** Assad Shiti, Idit Goldfracht, Naim Shaheen, Stephanie Protze, Lior Gepstein

**Affiliations:** 1 Sohnis Research Laboratory for Cardiac Electrophysiology and Regenerative Medicine, the Rappaport Faculty of Medicine and Research Institute, Technion‒Israel Institute of Technology, Haifa, Israel; 2 McEwen Center for Regenerative Medicine, University Health Network, Toronto, ON Canada; 3 Cardiology Department, Rambam Health Care Campus, Haifa, Israel

**Keywords:** Cardiovascular biology, Heart development

**Replying to** Christ et al. *Nature Communications* 10.1038/s41467-021-21949-z (2021)

In our recent report^1^, we combined developmental biology-inspired differentiation strategies of human pluripotent stem cells (hPSCs) to derive chamber-specific cardiomyocytes^2^ and a collagen-hydrogel-based tissue engineering strategy^3^ to generate ring-shaped ventricular and atrial-specific engineered heart tissues (EHTs). Detailed molecular, ultrastructural, and functional phenotyping, together with targeted pharmacology, confirmed the chamber-specific identity of the atrial/ventricular EHTs, and demonstrated the potential of these models for disease modeling and drug testing applications. The latter included the ability to induce reentrant arrhythmias in the atrial EHTs and the ability to terminate such arrhythmias with established anti-arrhythmic agents (flecainide and vernakalant). In the accompanying comment, Christ et al.^4^ raise concerns with regards to the relative immature properties of the chamber-specific EHTs and their different response to some of the anti-arrhythmic drugs tested (vernakalant and lidocaine) in comparison to their reported effects in adult human atrial and ventricular heart tissues.

The first point raised by Christ^4^ relates to vernakalant, a multichannel blocker (that also blocks the atrial-selective ionic currents *I*_Kur_ and *I*_KAch_), which is approved in the EU for acute conversion of atrial fibrillation (AF)^5^. In Goldfracht et al.^1^, we noted significant prolongation of APD_90_ values following vernakalant administration to atrial EHTs. Christ et al.^4^ refer to two studies by Wettwer et al.^6,7^, in which atrial trabecula/myocytes isolated from patients undergoing open-heart surgery were studied. In one study, they noted that vernakalant administration did not lead to APD_90_ prolongation in isolated atrial trabecula^6^. In their second study^7^, they suggest that this lack of APD prolongation stems from the inability of *I*_Kur_ blockade to prolong APD_90_ due to indirect activation of *I*_Kr_.

To address the aforementioned comment, we first aimed to reproduce vernakalant’s APD-prolonging effects in a different hPSC line and using a different experimental model. To this end, we evaluated the effects of vernakalant in a two-dimensional human-induced pluripotent stem cell (hiPSC)-derived atrial cardiomyocyte cell sheet model^8,9^. As shown in Fig. 1, vernakalant also significantly prolonged APD values in this 2D hiPSC-based atrial tissue model. These results were further corroborated in the very recent publication of Gunawan et al.^10^.

**Fig. 1:**
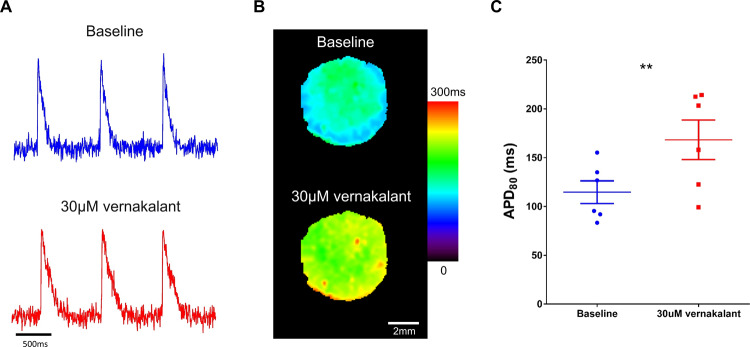
Effect of vernakalant on APD in hiPSC-derived atrial cell sheets. **A** Example of optical action potentials acquired from the two-dimensional hiPSC-derived atrial cell sheets at baseline (top) and following treatment with 30 µM vernakalant (bottom). Notice the atrial-like triangular-shaped optical action potential morphologies and the longer APD after the drug treatment. **B** APD_80_ color-coded maps depicting APD_80_ values at each pixel of the atrial cell sheets at baseline (top) and following vernakalant treatment (bottom). **C** Summary of mean APD_80_ values at baseline and upon administration of 30 µM vernakalant. Note the significant (**P = 0.0074, two-sided paired *t* test, *n* = 6) APD_80_ prolongation (from 115 ± 12 to 168 ± 20 ms) following the drug treatment. Source data is provided as a Source data file.

We also noted in Wettwer et al.^6^ that although vernakalant did not alter APD in atrial trabecula, this finding was limited to patients with sinus-rhythm. In patients with chronic AF, however, vernakalant significantly prolonged APD_90_^6^. This finding also correlated with the second Wettwer paper, where pharmacological blockade of *I*_Kur_ with either 4-aminopyridine or AVE0118 shortened APD in atrial cells from sinus-rhythm patients, but prolonged APD in AF patients^7^.

Interestingly, as described in our study^1^, in contrast to the ventricular EHTs, which displayed a normal activation pattern (“sinus-rhythm-like”), the vast majority of atrial EHTs displayed continuous fast and irregular arrhythmogenic activity due to multiple reentrant circuits (“AF-like”)^1^. This arrhythmogenic activity persisted for weeks but could be terminated, at least temporarily, by electrical cardioversion to allow the drug studies. Hence, one may consider the state of the atrial EHTs more analogous to the atrial cells obtained from the AF, rather than the sinus-rhythm, patients in the Wettwer study^6^. Consequentially, vernakalant’s APD-prolonging effects in the atrial EHTs might be in agreement with the human heart tissue studies^6,7^. These results may also open the road to potentially use this model to study certain aspects of atrial remodeling associated with atrial tachyarrhythmias.

The mechanisms underlying vernakalant-induced APD prolongation in the atrial EHT model was not studied. One option may be the known *I*_Kur_ blocking effect of vernakalant, which as described in Wettwer et al.^7^ can also prolong APD in AF patients. Christ et al.^4^ insightfully raised additional possible mechanisms, including the blockade of a potential basal activity of the *I*_KAch_ current or the potential for a reduced repolarization reserve in the hPSC-derived atrial cells, as described for hPSC-derived ventricular cells^11^.

In our study, vernakalant was able to terminate arrhythmias in atrial EHTs and prevent their immediate reappearance. These results are in line with multiple clinical studies showing vernakalant’s efficacy in terminating AF^5^. We did not further evaluate the mechanisms underlying vernakalant’s success in terminating arrhythmias in the atrial EHT model. The APD-prolonging effect (through increase in refractory period) may contribute to this therapeutic effect, but additional contributing factors, such as vernakalant known ability to induce a frequency- and voltage-dependent *I*_Na_ block may also play a significant role, since it preferably effects atrial conduction at fast rates.

The second point raised relates to vernakalant’s effects on ventricular repolarization. Vernakalant-induced changes in APD_90_ values in the ventricular EHTs were small (~10% rise from 403 to 445 ms) relative to the large increase (~93%) in the atrial EHTs. Nevertheless, based on the suggestion made by Christ, we reanalyzed our data, using a pairwise statistical comparison, and noted that this small increase was statistically significant. Whether this small change, which correlates with the ~20 ms QT interval prolongation reported in clinical studies^5^, is relevant to the safety of vernakalant is not known. It should be mentioned that the FDA’s recent rejection to approve vernakalant use was not related specifically to QT prolongation, but rather to other adverse events (hypotension, reduced contractility, bradycardia, conduction abnormalities, etc.) and the inability to predict patients at risk^12^.

The third point raised by Christ^4^ relates to the effect of the class Ib anti-arrhythmic agent lidocaine on the chamber-specific EHTs. Lidocaine application slowed conduction in both the ventricular (by ~66%) and atrial EHTs (by ~25%). However, the study in the atrial EHTs was probably not powered enough to identify statistical differences. It is possible that increasing the number of experiments (beyond the current five data points that are not normally distributed) would have resulted in the statistical significance. Consequentially, we agree with Christ^4^ that the absence of lidocaine-induced conduction slowing should not be used as a signature of an atrial tissue phenotype. Nevertheless, it is interesting to note that the greater conduction slowing observed in the ventricular EHTs correlates with the clinical use of lidocaine in treating ventricular, but not atrial, arrhythmias.

Finally, we agree with the general notion that the relative immaturity of hPSC-derived cardiomyocytes (hPSC-CMs) in terms of their molecular, ultrastructural, metabolic, contractile, and electrophysiological properties remains a major challenge in the field. In this respect, it is important to note that the relatively immature cellular electrophysiological properties referred to in our study (depolarized RMP, slow AP upstroke) were measured from single-cell, early-stage, chamber-specific hPSC-CMs prior to their use for creation of the EHTs. It is possible that both the prolonged culture time, as well as the 3D engineered tissue environment and mechanical conditioning can induce a certain degree of maturation, as previously described^13^. Although patch-clamp recordings were not repeated after EHT creation, indirect evidence, such as the response to Na^+^ channel blockers and the somewhat improved conduction properties, may point to such a process. Ongoing efforts in the field are geared toward the development of strategies to induce the maturation status of hPSC-CMs by using combinational hormonal treatments^14,15^, optimizing extracellular matrix composition^16^, mechanical and electrical training^13^, and inclusion of non-myocytes cardiac cells, such as fibroblasts and endothelial cells^17^. Such strategies can be used in the future to also promote the maturation the chamber-specific cardiomyocytes.

In conclusion, we thank Christ et al.^4^ for raising these issues that helped us clarify some of the results of our study and raised awareness for the need for critical interpretation of the new model. Like any new model, the recently described chamber-specific EHTs models^1,18,19^ possess advantages and shortcomings, which need to be recognized for optimal model utilization and accurate interpretation of the results.

Importantly, the chamber-specific EHTs represents an unprecedented opportunity to perform high-throughput drug screens to identify potential new treatment drugs for AF, which are eminently needed. These candidate drugs can then be further validated using adult human cardiac tissue, as suggested by Christ et al^4^. We and others will continue to improve the hiPSC-based cardiac tissue models, for example, by advancing its maturation status^13,18,19^, by introducing chamber-specific anatomical features (using organ decellularization or 3D bio-printing), and by including supporting non-myocytes^17^, such as cardiac fibroblasts and vascular cells to develop more clinically relevant multicellular tissue models. Introducing fibroblasts, for example, may allow to evaluate the role of cardiomyocyte–fibroblast interactions and, if stimulated, also of fibrosis in AF. We hope that the current work along with the discussion raised by the commentary of Christ et al.^4^, will advance the field forward, raise new questions and challenges, and facilitate further progress.

## Methods

### Generation and mapping of hiPSC-derived atrial tissues

A hiPSC-derived atrial cardiomyocyte cell sheet model was prepared, as previously described^8,9^. Briefly, differentiated hiPSC atrial cells were seeded as dense 20 µl droplets containing ~700,000 cells on Matrigel-coated 35 mm plastic dishes. The resulting atrial cell sheets were cultured in 2 ml RPMI/B27 medium. At days 5–10, specimens were loaded with the voltage-sensitive dye FlouVolt and studied using an EM-CCD-based optical mapping system.

### Statistical analysis

GraphPad Prism6 was used for statistical comparisons. Continuous variables are expressed as mean ± SEM. Paired *t* test was used to compare the effects of Vernakalant on the hiPSC-derived atrial cell sheets (Fig. 1) and on the ventricular EHTs (reanalysis of the data in Goldfract et al.^1^). *P* < 0.05 was considered statistically significant.

### Reporting summary

Further information on research design is available in the Nature Research Reporting Summary linked to this article.

## Supplementary information

Reporting Summary

## Data Availability

Source data are provided with this paper.

## Source data

Source Data

## Code availability

Data are collected and analyzed using a custom-designed software (kindly provided by Prof. Bum-Rak Choi, Brown University).
